# To wish you well: the biopolitical subjectivities of medical crowdfunders during and after Aotearoa New Zealand’s COVID-19 lockdown

**DOI:** 10.1057/s41292-021-00251-7

**Published:** 2021-09-22

**Authors:** Susan Wardell

**Affiliations:** Social Anthropology Programme, University of Otago, 2nd Floor Richardson Building, Leith St, Dunedin North, 9016 Otago New Zealand

**Keywords:** Crowdfunding, COVID-19, Narrative, Subjectivity, Health, Deservingness, Care

## Abstract

Crowdfunding platforms apply a marketized, competitive logic to healthcare, increasingly functioning as generative spaces in which worthy citizens and biopolitical subjects are produced. Using a lens of biopower, this article considers what sort of biopolitical subjectivities were produced in and through New Zealand crowdfunding campaigns during the 2020 COVID-19 lockdown. It focuses on a discursive and dialogical analysis of 59 online medical crowdfunding campaigns that were active during lockdown and chose to mention the pandemic. These pages pointed to interrelated biological, social and economic precarities, speaking to questions about how citizens navigate uneven needs during uncertain times. Findings showed that crowdfunders referred to the pandemic in order to narrate their own situation in culturally coherent ways and to establish context-specific relations of care. This included contextualising their needs through establishing shared crisis narratives that also made the infrastructural contexts of healthcare visible and performing relational labour in ways that aligned with nationally specific affective regimes. By highlighting their own vulnerability, crowdfunders strategically mobilised broader lockdown discourses of self-sacrifice on behalf of vulnerable people. In this way, New Zealand’s lockdown produced subjectivities both drawing on wider neoliberal moral regimes and specific to the nuanced and emergent moral systems of pandemic citizenship.

## Introduction

Studying online medical crowdfunding can offer a way to analyse healthcare systems and contexts, that is “informed by precariousness” (Paust 2020, p. 87) and also set in specific historical and national contexts. Recent years have shown donation-based forms of crowdfunding, for healthcare and other basic needs, are becoming increasingly institutionalised in many countries (Berliner and Kenworthy 2017). Both the outcomes and processes of crowdfunding form significant points of critical analysis for social scientists. The competitive logic of online crowdfunding requires participants to engage in significant labor, marketing their health needs and performing ‘deservingness’ to diverse audiences of potential donors. Because of this, recent literature acknowledges crowdfunding platforms as “generative spaces in which worthy citizens and biopolitical subjects are produced” (Paust 2020, p. 9).

In this article, I consider medical crowdfunding in the context of the coronavirus pandemic, as it first reached New Zealand in early 2020. Specifically, I examine practices of crowdfunding during and directly after New Zealand’s first national COVID-19 lockdown, which I take as a significant biopolitical intervention, with varied and widespread impacts on health and wellbeing, beyond the prevention of infection. I focus on campaigns for health needs not related to having contracted COVID-19, but which nevertheless chose to mention the pandemic as part of their wider illness narratives. I was interested in how making lockdown part of their stories had shaped specific ways of performing of need of vulnerability, and the way it had changed how moral deservingness was established and embodied. As such, I explored the following question: *What sort of biopolitical subjectivities were produced in and through New Zealand medical crowdfunding campaigns during the COVID-19 lockdown?*

To answer this, I analysed 59 New Zealand crowdfunding campaigns^1^ that were active in June 2020, on Givealittle and GoFundMe, as the main two platforms used for donation-based crowdfunding in New Zealand at the time. Although none of the campaign recipients in this dataset had COVID-19, the campaigns evoked the scope and complexity of the crisis (both the pandemic and the government’s response to it) through narrating the entanglements of their own health status with wider economic, political, bureaucratic, legislative systems, both local and global. But beyond this, it became part of the moral system within which they were seeking to meet their care needs. I thus use a discursive and dialogical analysis to examine how crowdfunders^2^ drew from nationally specific political discourses (and affective frames) associated with the lockdown as part of establishing deservingness in a culturally coherent way. This included performances of kindness, positivity and teamwork and forms of relational labor made possible or necessary through the shifting and certain structural context of healthcare and of wider social life. Ultimately, the study illustrates the way New Zealand’s lockdown generated new forms of biopolitical subjectivity that both drew on existing neoliberal moral regimes and appropriated lockdown discourses of responsibility, vulnerability and sacrifice.

## Background: Aotearoa New Zealand’s lockdown

New Zealand confirmed its first COVID-19 case on 28th February 2020. Amidst devastating effects already visible in other countries, the government quick to commit to an ‘elimination’ strategy—an approach only possibly because of the unique geo-political context of the isolated nation. A four-tiered alert system was introduced on March 21st, and on March 23rd Prime Minister Jacinda Ardern announced that in 48 h the country would enter a “Level 4” lockdown (Long et al. 2020, p. 12). This involved a system of nation-wide and rapid regulatory controls for the purpose of lowering population mortality in the face of a predicted wave of COVID-19 deaths. Key restrictions at Level 4 included the closure of all education facilities, all businesses except for “essential services”, cancellation of all gatherings, and people instructed to stay home in established household ‘bubbles’ at all times except for when travelling for medical assistance, grocery shopping (advised to be one person, once a week), or exercise within their local areas and with only members of their own bubble (Long et al. 2020, p. 13). As part of the protocol for reducing all non-essential contact, and to reserve space for the potential influx of COVID-19 patients, healthcare providers (including hospitals, emergency departments and general practices) were required to restrict access to their onsite facilities and staff, and make substantial changes to the delivery of routine services (Robinson and Wardell 2020). New Zealand spent 33 days in Level 4 lockdown, and another 16 days under the slightly eased restrictions of Level 3. The country moved to Level 2 on 13th May, and finally to Level 1 on 8th June, 80 days after the alert system was first introduced.

Over this period the government worked hard to build consensus around lockdown measures through national rhetoric emphasising solidarity and unity. This was ultimately successful in both high levels of public compliance, and broad uptake of positive affective regimes (Trnka 2020b). The early and strict lockdown protocol proved effective in stemming the virus’ transmission and reducing the impact of COVID-19 infections on the population of New Zealand, and amidst global praise the public also expressed pride in their shared efforts (Deguara 2020). However, nine months later, it is clear that the wider impacts of these decisions—including job losses, disruptions to education, and mental health stresses—fell across the population unevenly. Many countries report COVID-19 as exacerbating existing social inequalities (Paust 2020). In New Zealand, the effects of the lockdown varied along the axes of class, ethnic identity, and gender, as well as household composition (Trnka (2020b). I contend that it also varied on the basis of pre-existing states of health. People who were already unwell, or fell unwell during the pandemic with non-COVID-related issues, reported being impacted by many changes to the healthcare system—including delays or cancellation to surgeries, transplantations, or other treatments, restricted visitation and social support, a shift to virtual healthcare consultations, and reduced or postponed diagnostic services (Robinson and Wardell 2020). Confirmation of significant disruptions to cancer diagnosis, and some disruption to cancer treatment, have been made (Ministry of Health 2020). Impacts on psychological wellbeing were also measurable (Every-Palmer et al. 2020). In addition many wider social issues that link to wellbeing—including food insecurity, addiction, and housing—were exacerbated by the lockdown (Henrickson 2020). As non-COVID-related health needs continued, and in some cases worsened, under lockdown restrictions, many people simultaneously faced loss of income, and restrictions to many common mechanisms of fundraising (Robinson and Wardell 2020). Yet the internet remained a viable way for private citizens to tell their stories and solicit support.

### Connecting to crowdfunding

The process of online crowdfunding involves making a public call for financial contributions towards a project, goal, or need that is outlined on a web-based campaign page. These pages, customised by users, are embedded in a crowdfunding ‘platform’ or website, which provides the digital infrastructure for giving/receiving donations, and messages, and for sharing the page across a variety of networked social media platforms. Three main types of crowdfunding exist, including investment-based, reward-based, and donation-based. Within the donation-based sector, health or medical crowdfunding is by far the most common fundraising category—making up about a third to a half of all campaigns.^3^ Globally, GoFundMe, is the leading global crowdfunding platform, with 80% of the market share (Kenworthy 2019). GoFundMe make profit by taking a percentage of all donations. In New Zealand, a national non-profit crowdfunding site called Givealittle hosts the majority of donation-based campaigns, with GoFundMe the next most common choice.

A small body of literature on medical crowdfunding exists, and is growing—though not as quickly as the phenomena itself, which is already so ubiquitous as to sometimes appear ‘mundane’. This scholarly work is important for unpacking the ways this widespread phenomenon is situated in particular national, socio-economic and political systems, including its relationship to the neoliberal economic reforms that impacted many public healthcare systems in the 1980–1990s (see: Kenworthy 2019; Lee and Lehdonvirta 2020; Bassani et al. 2018). Both global and national comparisons show that high crowdfunding rates typically correlate with failures in public healthcare coverage and gaps in formal safety nets (Bassani et al. 2018; Kenworthy et al. 2020). In the USA, medical crowdfunding is prolific in the context of rising healthcare costs and debts, and inadequate healthcare insurance coverage, including systems to exclude those with pre-existing conditions (Kenworthy 2019; Kenworthy et al. 2020; Merid 2019); however, it is also common under Canada’s ostensibly ‘universal’ healthcare system, where long waits or specific unfunded treatments, as well as wider social support issues, are described as contributing factors (Snyder et al. 2017; Lukk et al. 2018). Only one other study (Neuwelt-Kearns 2020) has addressed crowdfunding in Aotearoa New Zealand—a nation that was also an early adopter of neoliberal policy, and yet has a robust public healthcare system by international standards and good health outcomes compared to most OECD countries. New Zealand maintains an extensive state accident compensation system, free hospital care for citizens or permanent residents, free (charitably funded) emergency services, and government subsidised primary care and medications, and nevertheless shows high rates of medical crowdfunding (Neuwelt-Kearns et al*.* forthcoming). This provides an intriguing point of comparison to other national contexts, and raises questions about the specific (rare, expensive, overseas, or experimental) medical costs that *aren’t* funded, as well as the wider social needs (i.e. housing, transport, childcare, income support) which are entangled with individual and family vulnerabilities during periods of illness.^4^ Indeed, nearly a quarter of New Zealand medical crowdfunding campaigns seek help with general living costs rather than (or alongside) direct medical costs (Wardell 2020). But while crowdfunding can be seen as responding to ‘gaps’ in social safety nets, not all campaigns are ultimately successful, and the overall effect is a worsening, rather than evening out, of existing social inequalities.

Deservingness—a key theme in the medial crowdfunding literature, as in wider studies of healthcare systems (Willen 2012)—is a key mediating factor in this. Crowdfunding platforms rely on positioning people as deserving of help, and yet some illnesses are easy to market, while others are stigmatised. The gender, race, and age of the recipient have been shown to be predictors of success, just as much as degree of need (Neuwelt-Kearns et al*.* forthcoming; Kenworthy et al. 2020, Wardell 2020). The ability of campaigners to align themselves with culturally specific value-such as being cheerful, independent or ‘hard-working’ also have significant impact (Gonzales et al. 2018; Paulus and Roberts 2018). Media literacies are thus key the conveying deservingness (Berliner and Kenworthy 2017; Neuwelt-Kearns 2020), with crowdfunding sites rewarding those who “learn to tell the story of their illness” in just the right measure (Gonzales et al. 2018, p. 3), and have the time and skill to put into doing this.

The theoretical frameworks applied to the study of crowdfunding to date have been limited, especially in terms of exploring subjectivities and social ‘becoming’. Kenworthy (2018) usefully applies a lens of affect to think about entanglements between North American donors and recipients in the global South, as does Kneese (2018) in analysing some highly politicised funeral campaigns, which required creators to consider an imagined audience, and social contexts, as well as the specific mechanisms of the platform. Gonzales et al. (2018) draws on the framework of ‘identity shift’ to consider the dynamic relationship between public self-presentation to audiences, and to consider how this may enact a change in user identity (through internalising these as part of the self). A framework of biopower has yet to be systematically applied to medical crowdfunding, and yet I argue it has considerable potential to connect to and extend existing literature, by highlighting the inequality of the “social conditions” in which health-related identities, or subjectivities, are formed (Whyte 2009, p. 6). In this study, it provides a useful way to consider what the illness narratives shared through crowdfunding platforms reveal about biopower, deservingness, and care relations within the social conditions of lockdown.

### The biopolitics of lockdown

New Zealand’s COVID-19 lockdown was a clear example of the exercise of biopower in the governance of “life itself”. As a system of rationalised regulatory interventions on collective existence, deployed at the level of the nation “in the name of life and health”, and leveraging the scientific authority of a number of key public figures, it lines up closely with the characteristics of contemporary biopolitics described by Rabinow and Rose (2006).

As a democratic nation currently led by a Labour (centre left) government, New Zealand’s lockdown relied not on militaristic powers of enforcement but on consensus building and collaboration between citizens and the state. Through daily appearances at both formal press conferences and informal livestreamed question sessions, Prime Minister Jacinda Ardern worked hard to “transform the affective landscape of the lockdown” with positive rhetoric (Trnka 2020a). This strategy of “microregulation of a national community at the emotional level” during crisis aligns with historical civil defense protocol in the US, which aimed to shift the public from potentially paralysing emotions of fear, into more functional affective states (Masco 2008, p. 368). Both evidence of this, and a contributing factor to it, the public responded to Ardern’s rhetoric via a number of grassroots movements (later endorsed by the Prime Minister) that provided an acceptable outlet for positive action and an expression of solidarity while physically distant; including the ‘teddy bears in windows’ movement, as one example. It is therefore important to consider, as Trnka (2020a) urges, the public’s role not only as objects of state power, but as subjects actively partaking in biopolitical regimes. I use the findings from this study to suggest that new forms of subjectivity were generated during this time, through these and other practices of pandemic citizenship..

Subjectivities can be approached by analysing the symbolic forms people use to represent themselves, to themselves, and to one another (Biehl et al. 2007, p. 7). While many aspects of public life were interrupted during lockdown, social media remained active as a site for self-reporting on experiences of the pandemic at an intimate, everyday level. On these platforms, and as part of mediatized performances of health and illness, subjectivities are clearly evident as situated in biologies, as well as having a cultural specificity, political location^5^and economic position (Kleinman and Fitz-Henry 2007, p. 53). The immaterial labour of visual and written storytelling on social media generally (Rose and Spencer 2016) and on crowdfunding platforms specifically, remained as evidence of ways of being amidst a state of emergency, but also evidence that subjectivities are dynamically formed and transformed by individual and relational practices that can occur online as much as face-to-face.

It is important to emphasise my position that crowdfunding platforms constitute an arena in which subjectivities are not only made visible, but in fact *made.* Amidst the COVID-19 crisis, these sites stood as a place where individuals (and their loved ones) worked on themselves “in relation to truth discourses, by means of practices of the self, in the name of individual or collective life or health” (Rabinow and Rose 2006, pp. 203–204). Their campaign pages can thus be read as *technologies of the self, performing a reflexive, semi-confessional role*—*a tool for both self-declaration and self-cultivation. As such, the campaigns that mentioned the pandemic not only reflected but helped constitute ways of being in the world specific to that time and context.* The contribution of this research is, therefore, to show donation-based crowdfunding platforms as one arena of subjectification, during the exercise of biopower that was New Zealand's lockdown. But also, to use the framework of subjectivity to consider the agency of crowdfunders, who were not only ‘working on themselves’ through these campaigns but were also ‘making it work for themselves’ as a strategic means of soliciting care, even amidst a much bigger national health crisis.

### Crowdfunding during COVID-19

Crowdfunding has featured in the pandemic in striking ways, and in many different places. Crowdfunding has been used to fundraise for PPE and other medical equipment, including by researchers and scientists in Ireland (Rowan and Laffey 2020); NHS doctors in the U.K. (Sayburn 2020); and various high-profile individuals (Bienvenu 2020). Individual citizens as well as community/charitable groups have turned to crowdfunding as an alternative ‘safety net’ to cover general life expenses (food, rent etc.), or to keep small businesses or cultural institutions afloat (Popper and Lorenz 2020; Cole 2020; Wakui 2020, p. 317). In March 2020, just as New Zealand began lockdown, GoFundMe was reported to be “facing the greatest demand it has seen since its founding in 2010” (Popper and Lorenz 2020, n.p.), with the number of coronavirus-related campaigns jumping by 60% (from 22,000 to 35,000) in just four days (Popper and Lorenz 2020, n.p.). GoFundMe also started their own lucrative “COVID-19 Relief Fund”, gathering $356,469 between March 12th 2020 and August 3rd 2020, from 47,000 donors (GoFundMe 2020). New Zealand’s own platform, Givealittle, had two pages dedicated to COVID-19 (Givealittle 2020b). The ‘COVID-19 Support’ page hosted links for a variety of individual and community fundraisers, including fundraisers for small businesses and tourist-reliant attractions, PPE and other medical equipment, specific vulnerable communities in NZ or overseas. The ‘COVID-19 Charities’ page listed campaigns supporting charities which, due to the pandemic, had to cancel or change their normal fundraising events (Givealittle 2020a).

Despite the wider impacts on the country’s economy and on many individual livelihoods, Givealittle reported that crowdfunding donation figures ultimately went *up* over this time, reaching $2.82 million in April: a number “well above the usual $1.9 m monthly donations” (Perpetual Guardian 2020, p. 5). Similar trends in altruism were recorded across three French crowdfunding platforms, according to Moine and Papiasse (2020, n.p.). These researchers assert that studying crowdfunding trends during a crisis like COVID-19 can provide insight into collective needs, anxieties and “behavioural responses” (Moine and Papiasse 2020). I emphasise that it can also become an intimate window into storytelling and subjectivity, with high stakes.

## Methods: studying crowdfunding stories and practices

Many people learn to tell the story of their illness via the internet (Gonzales et al. 2018). Illness narratives have been of interest to medical anthropologists and sociologists for a long time, and in recent years many researchers have studied health stories and health identities through examining online texts, places, and communities, as sites of situated social performances. Crowdfunding platforms provide a scaffold for storytelling, via an interface which allows users to customise their own publicly visible campaign page with text, photos or video, and supports them to write regular written updates and public responses to individual donors. The sites also provide instructional pages on how users can create “compelling, multi-media narratives about their illness experiences” —with the aim of increasing donations, and thus also company profit, in the case of GoFundMe (Kenworthy et al. 2020, p. 24; Berliner and Kenworthy 2017). Their tips often focus on selecting attractive photos or adopting a lively writing style. But there are more nuanced socio-moral questions around how to tell one’s illness story successfully, especially during a time when the audience is also potentially distracted by the massive social shifts of a global pandemic.

To explore these complex dynamics in a nationally specific context, I analysed 59 medical crowdfunding campaigns made by New Zealanders, or for people in New Zealand, and that mentioned COVID-19 in either the main story or the updates. This dataset was taken from a larger dataset of 573 campaigns identified as part of a longer, multi-methodological, study of medical crowdfunding in Aotearoa New Zealand (Marsden Fast-Start Grant, “Online Medical Crowdfunding in New Zealand: Illness, giving, and moral emotion”, PI Susan Wardell 2020–2023). The first phase of the project focussed on producing a snapshot of the landscape of medical crowdfunding in New Zealand—including who was fundraising for what. The sample was taken from campaigns active on Givealittle and GoFundMe between 8 and 19th June—directly after New Zealand had moved down to Level 1 of lockdown protocol. Only campaigns by and for private citizens were included, with fundraisers for organisations, or organisational campaigns, excluded. The 573 campaigns fitting these criteria were coded for 30 different variables by four different coders. It soon became apparent that a number of campaigns were mentioning COVID-19, and I suggested at this time that we (myself and my research assistants) record all references to the pandemic. It was this subset of campaigns that I returned to later in the year.

Of the 59 campaigns that mentioned COVID, most were for New Zealanders based in New Zealand (43), while some of the remaining were run by New Zealand residents for overseas family, and some for New Zealanders based overseas. The majority of the campaigns related to illness, as was the norm for medical crowdfunding overall in New Zealand (Wardell 2020), and a high number of these (23) were for cancer. Also in line with wider national crowdfunding patterns, not all requested funds were designated to cover medical services, equipment, or consumables directly, however, but more often outlined a need for help with general standard of living costs (such as rent, bills, food, and caregiving costs), travel for treatment, and accommodation.

After recording the general campaign features, and reported impacts of lockdown in each story, I analysed the 59 COVID-19-related campaigns using a discursive and dialogical approach. For this I considered the written text of the main campaign page, photos, and updates—all part of the overall campaign ‘narrative’—with all campaigns viewed embedded in the visual context of the platforms’ interfaces rather than as extracted components. While most of the campaigns had commenced before COVID-19, I focussed on campaign material from during lockdown, considering this in the context of the wider campaign narratives. An iterative, open coding process was undertaken—this being ‘discursive’, after Foucauldian traditions of critical discourse analysis (i.e. with attention to the relationship between language used, institutions, and systems of expert knowledge), and ‘dialogical’ in the sense of considering addressivity, ‘voice’, and speech acts (things being *done* by the text, as well as things being communicated by it).

The first layer of coding for these campaign narratives generated a code list which was then organised into the following themes: *Making systems visible; Temporality; Emotion codes for uncertainty; Weighing cost/risk; Performative kindness; Team of 5 million; Relational labour; Experts on isolation; Independence and resilience; Dependence and need; Constraints outside oneself; Money in lieu of presence*. The relationship between these themes was considered, and they were re-organised under two main findings discussed in the follow section.

The study functioned under approval from the University of Otago Human Ethics Committee (reference code 20/028). All data used for this phase of analysis were publicly available. However, as I take quotes out of their original context and purpose on the platforms, I have chosen to remove the names of individual crowdfunding recipients and not to provide direct campaign links.

There are limits on the empirical reaches of this data. It does not provide a *direct* window into lived experiences during COVID-19, nor provide a systematic review of the structural changes of the healthcare system of this time, as the accounts provided represent a relatively small sample of New Zealand citizens engaging with healthcare systems at this time, and are subjective in nature. However, studying crowdfunding campaigns can provide insights into this period of time in a different way. Considering the structural contexts of this symbolic arena (including its economic prerogatives) it should be regarded as a performative space, and a social one. Analysing crowdfunding narratives thus provides a novel way to respond to Trnka’s (2020b, p. 13) plea that scholars continue to “interrogate the public’s affective engagements with the state, assessing the collaborative construction of notions of collective responsibility, obligation, community and care”. It reveals how people marketed complex health needs—including those created by government decisions—amidst a period of intensified uncertainty and tight public adherence to biopolitical regimes.

## Findings and analysis

Scholarly writing on states of emergency have discussed the rapid expansion of state power based on a ‘crisis imaginary’ (Trnka 2020b). By March 2020 the media, replete with images of death and grief overseas, had already stirred a crisis imaginary in New Zealand. Not all medical crowdfunders during this period chose to mention COVID-19 in their campaign stories. However, those that did contributed to ‘envisaging’ (Roitman 2014) New Zealand’s pandemic lockdown. Yet there is a level of impossibility, according to Roitman (2014), in representing crisis, and crowdfunders grappled with the doubly challenging task of working out how to tell their stories when their personal crises overlapped with a many-layered social crisis, and was sometimes even created by it (a problem I explore later).

In the follow section I organise my findings around the way crowdfunders worked to: (1) contextualise their need through establishing shared crisis narratives and making systems visible, and (2) embody moral deservingness by performing positive traits or values, and enacting relational labour to establish reciprocity. I use my data to show how both were done in ways specific to the context of COVID-19 and the New Zealand lockdown.

### Contextualising need through establishing crisis narratives

Crowdfunding campaigns present stories that have a particular setting. This is both evoked within the text and images of the user-generated narratives, and within the interface of the platform, where geospatial tags and timestamps help orient the audience by locating the illness stories in time and space (Paust 2020). Narratives often unfold in timestaped updated that follow on from the narrative on the campaign's main page, giving a prevailing sense of linearity, and revealing how and illness and its treatment, along with other aspects of individual/family circumstance, might develop over time. In the case of this dataset, portions of the narratives were also mapped against the wider social and spatiotemporal changes of New Zealand’s COVID-19 response, and specifically, the different levels of lockdown the country moved through.

#### The setting of ‘a scary world’

References to New Zealand’s COVID-19 response were sometimes quite explicit, with some campaigns mentioning the coronavirus directly and/or sharing detailed discussions of the specific effects of lockdown restrictions. In other campaigns, the pandemic was referenced more implicitly, as “everything happening in the world today” or “this time of isolation and uncertainty” or “a pretty scary time we are moving into”. Such comments functioned on the assumption of shared familiarity of the reader with the development of the pandemic, in New Zealand and globally. Many involved explicit emotion codes, e.g. “a lot of us are worried and scared”.

While illness experiences are often isolating, this approach created a larger narrative setting—the “scary world”—in which the recipient and audience were both present. Setting a potentially distancing narrative of personal crisis amidst a larger narrative of shared crisis, made the context of the need more tangible for the reader (and potential donor), while collective pronouns such as ‘we’ and ‘us’ assisted in creating an imagined community, interpellating the reader into that community, that shared story.

#### Time, waiting, and making biomedical systems visible

Time was evidently important to both illness and treatment in these narratives. Many of the campaign stories made visible the tightly tuned timing of the bureaucratic management of biomedical healthcare systems (as an assemblage of people, place, and technologies), by narrating moments in which these broke down or failed– a useful way that normally opaque infrastructures can be studied (Bowker et al. 2010). In this case, crowdfunding narratives made visible the normally taken-for-granted paths between diagnosis and treatment that make up “normal services”, by highlighting the consequences of their disruption. “It seemed like things were on track [for surgery]. Then COVID-19 swept through”, as one cancer patient wrote in their campaign. Temporality is key to the crisis narrative, and as in this example, many campaigns mentioned the pandemic to distinguish between normal time, and the more unruly (urgent, disrupted, and delayed) ‘crisis time’.

Waiting was also a common theme. In many narratives, experiences of waiting introduced time as a dimension of risk. In one campaign, a woman narrated how her sister received a biopsy in early March, and “A week after that COVID-19 took over the world and [she] waited and waited and waited” to be eventually told by phone a week later that the cancer had spread and she would be on life-extending measures only. Another person described how the wait changed their treatment entirely as it “took chemotherapy off the table”. In this, waiting links to urgency—a trope common in crowdfunding campaigns, often part of establishing the moral worth of a cause (Paust 2020). Affects of urgency are a part of the assemblage of emergency too (Anderson 2017), and many of the campaigns I analysed effectively referenced spatiotemporal dimensions of the lockdown to emphasise the urgency of the need. Some campaigns gave specific timeframes in order to do this, such as a New Zealand man living in the UK who explained needing a stem cell transplant to treat his myeloma blood cancer “within the next 10 weeks or sooner the better”. His campaign explained that the NHS services in the UK had shut down, and he could not wait for them to resume, so was fundraising for expensive and difficult-to-secure flights back to New Zealand, while “the clock is very much ticking.”

#### Distance, movement, and presence

Movement, borders, citizenship, and other geo-political factors affecting treatment access —— as well as access to wider social care networks —— were mentioned prominently in this dataset, with stories revealing the entanglements of contemporary biomedical healthcare with numerous global and local systems. “Borders were closing rapidly around us,” one person wrote evocatively of their efforts to return home to where they would have medical care access. Several campaigns narrated difficulty in procuring or transporting stem cells from overseas. In one campaign for a 29-year-old New Zealand woman with Acute Myeloid Leukaemia, her friends explain, “With the COVID-19 pandemic raging around the world, the task of getting the stem cells to [the recipient] created an additional challenge. Luckily, [her] new stem cells were frozen, and transported by airfreight, from Quebec to Singapore.” A young father needing a bone marrow transplant was unable to search worldwide “because of the virus” and had to find a donor within New Zealand instead, taking him “from billions of choices to just the donors of our five million population”. These stories are not necessarily common for the public to hear, nor are these aspects of biomedical infrastructure typically transparent—their politics more often “buried in technical encodings” (Bowker et al. 2010, p. 98)—yet they were deliberately unpacked by crowdfunders in order to contextualise their needs.

Many campaign narratives emphasised deprivation of certain forms of interpersonal care or support, due to restrictions on travel and visitation. Emphasis on the constraints on the audience enabled campaigners to put impetus on donating as one of the few remaining avenues of support. Indeed, the wording of some seemed to encourage monetary donations as a ‘stand-in’ form of care. For example, someone campaigning for a New Zealander trapped overseas by border closures rallied donors by suggesting: “We need to support them the best we can from here in NZ.” Another campaign organiser appeared to suggest their own labour of setting up and managing the campaign for a loved one, was a contribution in lieu of being there in person. A family member of another recipient wrote how much they appreciated reading the messages of support via the platform, given the physical or geographic separation they faced. Such separation is not a factor totally unique to the lockdown, as cross-national campaigns were common pre-COVID too. However, the significance of this was heightened in the context of lockdown’s limitations on hospital visits, travel, and social activity even *within* national borders.

#### Economic constraint, ‘unexpected’ needs, and dependence

Both global and national economic shifts due to COVID-19 were acknowledged in campaign narratives, as factors producing need. In crowdfunding, the connection between financial need and “unexpected” circumstances can help justify the dependence of the recipient on the crowd (Snyder 2016). Financial impacts mentioned included things as large as global currency fluctuations, as was described by a New Zealand-based campaigner who had been fundraising for 6 months before COVID began, for a specialised Multiple Sclerosis therapy only available in Germany. In an update in May, she explained that “Unfortunately the COVID-19 pandemic has reduced the value of the NZ dollar so the cost of treatment has increased by about $15,000 at this point, and due to flight restrictions we have had to postpone the treatment date a further 6 months”. Other impacts were more individual, and focussed on the capacities of recipients or their carers to meet costs, rather than the objective costs of their care.

Job loss was mentioned frequently and framed through the language of constraint: being “unable” to work, or “needing” to stop work or “having to” use up all their leave. The scale of COVID-19 job loss, and its relative lack of stigma compared to other forms of job loss, again helped paint the needy situation of the recipient as rising from temporary circumstances outside of their control—a more morally acceptable position than long-term dependence (Snyder 2016; Neuwelt-Kearns et al*.* forthcoming). The cancellations of fundraising efforts that required physical presence or social proximity, due to lockdown protocols, were also frequently mentioned as an economic impact of COVID.

The process of mapping out these changes in the narrative of the campaigns had the dual purpose of emphasising external constraints and highlighting the self-responsibility of the crowdfunders in ‘normal’ circumstances. As one man—stuck alone in the UK—wrote, “I am normally very self-sufficient and resourceful and given time and once I am reasonably healthy I should be able to sort myself out”. As others have noted, campaigners that focus on asserting independence and self-responsibility, feed into ideas of crowdfunded care as “a discrete intervention during abnormal circumstances that a market can solve, rather than a pervasive and necessary feature of social life” (Neuwelt-Kearns 2020, p. 83). In this situation crowdfunders evoking the exceptional circumstance of COVID work to shift not only their own moral positioning (from structurally dependent beneficiaries to temporary victims of a *shared* crisis), but also to shift the moral positioning of their potential donors, by distancing the practices of giving/receiving money from the idea of ‘welfare’, and aligning it instead with ideas of emergency aid.

### Performing relational labour, establishing reciprocity

Presenting a positive moral subjectivity—as someone deserving of care—involved a variety of components, established not only through narrating one’s actions and choices *to* the audience, but in actions undertaken with and *for* the audience. Evident in these campaigns was ‘relational labour’ (Baym 2015)—a form of unpaid social labour focussed on regular (in this case asynchronous) communication with audiences, in order to build social relationships, typically with a longer-term financial incentive, such as fostering paid work, supporting a career, or in this case, securing donations.

Crowdfunding audiences are, in reality, often ill-defined; a mix of friends, family, colleagues and acquaintances across existing social networks, and wider networked publics, including ‘strangers’. Despite this, the crowdfunding narratives I studied often addressed imagined and non-present others with tones of intimacy and care. Distinct phatic expressions within the text, and especially within updates, performed social actions in ways that drew directly from the context of the New Zealand COVID-19 lockdown and its tight set of political messaging.

#### Performing (and reproducing) kindness

The first common speech acts I observed were ‘checking in’ and ‘well-wishing’, with many efforts made in updates to enquire of the wellbeing of their audiences and to express care and positive wishes towards them. Examples from the period of level 4 and 3 restrictions included: “I would like to take a moment to wish you all well.”; “Please stay safe stay kind  and remember to check up on your loved ones.” and “I know things have been really rough for us all recently and my heart is with you.” Significantly, this echoed the Prime Minister’s own language of ‘checking in’ in her regular updates, as well as public health messaging about ‘checking in on the elderly or vulnerable” (Martin-Anatias 2020), and the repetitive emphasis on kindness and solidarity among a “team of five million” (Long et al. 2020, p. 15; Appleton, forthcoming). It is important to stress that the crowdfunders did not just *reference* kindness, but *enacted* it, through the speech acts involved in these written updates. This included showing understanding towards others’ limitations, for example: “We understand COVID-19 has impacted so many people in New Zealand and many may not be able to assist and it is a difficult time to be asking for any donations”.

Other speech acts included ‘rallying’ each other towards collective values or idealised affective states. “Kia kaha, be kind to each other.” “Be kind at all times through this extremely difficult period of time. We will get through it together but divided we will fall.” [emphasis mine]. Adding in the ‘scary world’ setting, one person stated that “COVID-19 is creating havoc in our world and a lot of us are worried and scared. We need to stick together (at home of course) and do what we need to do” [emphasis mine]. Notably, these comments relied on collective language. This sums up efforts to foster solidarity in the imagined community of New Zealand, with digital technologies providing a crucial tool for this socially reproductive labour, during the physically distanced period of lockdown.

#### Reciprocity through gratitude and gifts of wisdom

Expressing gratitude was another way campaigners observably performed relational labour. Gratitude is an established theme in crowdfunding campaigns (Paust 2020), and can be part of what is offered back to donors to try and establish a reciprocal relationship. For this reason, it sometimes blurred into other forms of relational labour like ‘checking in’ and ‘well-wishing’ in my data, and conducted in a similar affective register, for example: “you all supported me throughout the last few months and that is something I will never forget. Now is the time to take care of yourselves! Stay home if possible please. Take care.”

Sharing wisdom or expertise was another technique through which crowdfunders ‘gave back’ to their audiences in a manner also specific to the time/space of the lockdown. One crowdfunder had a painful, chronic condition (described as a “living nightmare”), which typically kept them at home and somewhat socially isolated, even before the pandemic. In several updates during lockdown, this person enacted checking in, expressing well wishes to his audience, and conveying gratitude for their support, before also suggesting his audience “take it from me” about how to cope with the situation of lockdown isolation. A different campaign, organised by the partner of a young woman with cancer, reminded the audience that they had been in near-complete isolation since January already, and had “learnt a few useful tips for how to pass the time and remain sane” that they wanted to share. Several paragraphs of numbered points followed. Another recipient—a father on his third ‘battle’ with cancer in eight years—also offered his own strategies on ways to mentally reframe situations of isolation, repeating several times that it is “only a simple piece of advice,” and he is “only offering some perspective,” but also that he hopes to help any worried and anxious people.

This practice of offering advice taps into wider ideas of suffering as leading to wisdom (Brady 2018), and yet the wisdom offered here is less spiritual and more practical, and with an applied COVID-specific context i.e. focussing on social isolation, waiting, and mobility restrictions. The practice as I observed it echoed other networked infrastructures of care, such as online mutual-support groups or illness blogs, through which ‘expert’ patients (and caregivers) contribute their own experiences and advice—sharing “affective survival strategies that enable the management of the emotional life of illness” (Merid 2019, p. 170). It can also be framed as an application of “crip wisdom” (Imhoff 2020)—i.e. the adaptive and embodied techniques of disabled or chronically ill individuals and communities, to deal with disabling social structures. Yet here the advice, while based in experiences of ill-health or disability, finds purchase as a resource for anyone living under lockdown conditions, (potentially) creating intimate connections note just within illness communities, but with wider publics. In this way, the lockdown allowed for factors that were usually social vulnerabilities to be ‘evened out’ by the new challenges and vulnerabilities other members of the public were experiencing at the same time. By positioning themselves as ‘experts’, unwell crowdfunders and their families countered the riskier moral position of being dependents only, by presenting themselves as people with something valuable to offer. This is all significantly contextualised by the *marketized* settings of the platform—where the advice offered is never more than an inch from the ‘donate’ button—differentiating it from other online care infrastructures by more directly commodifying this relational labour and embodied wisdom.

#### Transparency, accountability, and responsibility through risk management

Important in the context of rapid economic shifts, crowdfunders also laboured to perform financial transparency and accountability to their donors. This included one campaign that promised pictures of the alternative activities that were going to be undertaken, when a fundraiser marathon was cancelled due to COVID-19—also an example of the way the interface allows for using visual mediums for visibility and, in this case, accountability. Another campaign provided a written update explicitly entitled “Financial transparency update”, in which they explained exactly how they would break down funds received so far, as well as explaining the effect of several aspects of COVID-19 on their personal savings capabilities. These findings align with Neuwelt-Kearns’ (2020) study which emphasised the efforts that campaign organisers go to, to manage audience expectations when changes occur. Being responsible for not only adapting to COVID by managing one’s fundraising efforts, but also updating donors in detail, was one key form of responsibility being performed through these communications, but not the only one.

Medical anthropology literature has provided many examples of the ‘responsibilised’ patient—who is aware of and manages their own wellbeing—as highly valued amidst a neoliberal moral economy. This can involve navigating risks to one’s health, which is not always straightforward since some risks have to be weighed against others. The crowdfunders visibly performed the ‘responsible’ patient through stories of doing this. For example, one campaigner narrated their process of deciding against chemotherapy as “too high a risk” for a lowered immune system, during the time of a pandemic. Another with a chronic condition wrote about how they “Haven’t seen the nurse in 2 weeks because of COVID-19 and they don’t want to take the risk. Which is fair enough. I don’t want to take the risk either”—a comment seeming to balance a sense of deprivation or constraint, with a way of framing himself as a responsible, risk considerate citizen.

Risk becomes visible here as a “practicable entity” (Roitman 2014, p. 76), but also a multi-vectored one. In this context, the economic and biological are often entangled, such as for one family where two daughters, who lived with their ill mother, struggled to pay for care for her cancer. A family friend, who wrote the campaign, explained, “they can’t return to work until level 2 of the lockdown, but if they do return to work, there is a risk of passing on viruses to their mum…” [emphasis mine]. Risk and vulnerability—which included components of being *at risk*—were also closely linked, with another campaign emphasising the recipient being “unable to work as she was deemed to [sic] vulnerable”. Some evoked the experience of being ‘vulnerable’ quite evocatively: “He is one of ‘those’ people who would be taken out by COVID-19. For us it was like having a pack of wolves waiting outside the gate. We were so scared every time we needed to go beyond our home, for the many hospital appointments and medical supplies.” Many of these stories evidence ‘need’ as resulting entirely from both external constraint *and* responsible choice—all morally acceptable reasons for requesting help—but dire, nonetheless. Ultimately, it is the narration of the process of weighing risk that performs responsibility, but also reveals vulnerability (by hammering home the at-risk-ness that is being carefully managed). In the next section I unpack this further, discussing the tensions these campaigners were navigating, in how to be responsible, needy *and* deserving, that were specific to the times of COVID.

## Discussion

It is through the study of subjectivity that the moral comes into view; made visible through exploring the “inward reworkings of the world and the consequences of people’s actions towards themselves and towards others” (Biehl et al. 2007, p. 15). The findings of this study provide an illustration of how some biologically and economically precarious people within New Zealand in early 2020 reworked the world of COVID-19 into their personal narratives of illness, vulnerability, and constraint, and considers what this indicates in terms of the subject positions they were taking up. Studying the biopolitical subjectivities produced in their attempts to narrativise (and competitively marketise) their healthcare needs in an unprecedented context, can expand on other work on crowdfunding research that emphasises how campaigners draw on different vectors of deservingness to establish themselves and their cause as ‘morally worthy’. Specifically, it elucidates something of what moral deservingness looks like amidst a pandemic, and how this connects to sociality and the politics of care, in times of crisis.

### Sociality and care in the ‘team of five million’

The logic of the deserving subject is shaped by the imagined ties of “nationhood” (Neuwelt-Kearns et al. (forthcoming, n.p.)). In Aotearoa New Zealand, the pandemic constituted these ties in newly intensified ways. I suggest that COVID-19 transformed New Zealand into a “community of fate”—a term Peter Baehr coined to describe “a pattern of temporary social cohesion arising from a mass emergency”, based on his study of the SARS epidemic of 2003 in Hong Kong (2005, p. 181). Trnka et al.’s large-scale surveys of New Zealanders during the COVID-19 lockdown indeed found strong affective relationships between individuals and the nation, and individuals and the government, emerging over this period (2021). While the nation of New Zealand of course pre-existed COVID-19 as a biopolitical entity, and an imagined community, COVID-19 brought a new way of seeing and experiencing ourselves. This included new forms of biosociality shaped by awareness of collective vulnerability to disease communicability (discussed in the following section), and the construction of the 'team of five million' with a shared purpose, reconstituted in relation to crisis.

Symbolic constructions of this ‘team of five million’ occurred visibly through both official public health messaging and public and media discourses—focussed on unity and teamwork in a manner resonant with existing national values and identities (including the obvious link to sports culture). The medical crowdfunding campaigns I analysed offered a window into processes of subjectification of individuals as members of this team. Points of evidence in the findings include strong use of collective language, reference to shared experience, and enactment of team values through relational labour—all with the goal of evoking a particular response—of both caring *about* and caring *for—*on the part of audiences.

What form of social relations, and what relations of care, are made possible in this newly re-imagined community of fate? A care ethics lens is used across many disciplines to cultivate understandings of “how interdependence and relations of responsibility permeate social and political life” (Neuwelt-Kearns et al*.* forthcoming), and can help to elucidate this. At the mercy of diffuse networked publics to fund their *individual* needs, the findings above made clear how crowdfunders’ attempts to trigger relations of care were embedded in the COVID-19 setting. Conveying their individual needs as entangled with the ‘scary’ times, helped to mobilise a type of “productive fear” (Masco 2008, p. 368) among potential donors, that worked because of the shared belonging in the ‘community of fate’ associated with these times. However, there were tensions in the way the campaigners had to grapple with the costs and sacrifices of being a good team member themselves—including maintaining positivity and team spirit during reduced healthcare services and reduced social support resulting from lockdown policy compliances—while still seeking to have their own needs met.

While acknowledging *shared* suffering during the pandemic, and performing reciprocal care relations themselves, crowdfunders still, at some point, had to distinguish their needs or their suffering as greater than that of others. “[We] know that this would be a hard time for everyone facing a pandemic but…” [emphasis mine] stated one campaign, at which point, having performed kindness and understanding, they preceded to state their case. “Not everyone has an easy bubble to live in during this pandemic” another wrote, making an implicit comparison between the imagined audiences’ assumed privileges and their own situation. The comparison raises a question: if the goal of crowdfunding is “producing a worthy illness” (Berliner and Kenworthy 2017), it is significant to consider what illness or need might be considered ‘worthy’ compared to COVID-19 itself? After all, some of these campaigns were competing, in the ‘attention economy’ of social media, with a flood of coverage of COVID-19, including crowdfunding campaigns more specific to COVID. Framing their own needs as related to the pandemic, even indirectly, could thus be seen as a strategic alignment of ‘salience’ or newsworthiness, boosting themselves within hierarchies of deservingness, in an environment where attention could be converted into money.

Crowdfunders did seem to perceive ‘care’ as potentially spread thin in this context of COVID-19 as some of the above comments show, yet rates of donation did ultimately increase over the period of lockdown (Perpetual Guardian 2020). I suggest that this may be due, at least in part, to the successful subjectification of the wider population into the ‘team of 5 million’, with its emphasis on taking personal responsibility for the wellbeing of others. In particular this worked through an alignment between New Zealand’s national COVID-19 discourse and the subject positions of crowdfunders, around the category of ‘vulnerable people’, as I turn to explore now.

### Strategic vulnerabilities and networked responsibilities

Judith Butler writes that we are “constituted politically” by virtue of the social vulnerability of our bodies (2004, XII, in Trundle et al. 2018, p. 4). Her post-9/11 work posits a sociality “premised upon the threat of bodily harm” (*ibid*). COVID-19 lockdown protocols were also premised on a threat of physical harm to great numbers of people, emphasised in frequently circulated news stories and statistical models regarding what numbers of cases and death tolls might have been *if not* for the nation’s rapid adoption of the strict lockdown protocols (for example see: Daalder 2020; Cooke 2020). The virus highlighted, in new ways, our social vulnerability as well as “our collective responsibility for the physical lives of one another” (Butler 2004, 30, in Trundle et al. 2018, p. 14). In doing so it fostered a form of biosociality not just around/within one particular social group, but with the potential to function at the level of the nation, as the previous section has described. However, physical vulnerability during the time of COVID-19, as at any time, was not evenly distributed. Rather, uneven vulnerabilities to COVID-19 were highlighted early on, with elderly and immuno-compromised pointed out repeatedly in news media and political addresses as specific groups who were vulnerable (i.e. most at risk of harm), and who then became the specific ‘targets’, or beneficiaries, of both government and public responses. On behalf of these people especially, the population was asked to bear the ‘cost’ of stopping the spread of the disease through responsible adherence to the biopolitical interventions of lockdown.

An emphasis on the public health outcomes of civic duty, through lockdown compliance, was not presented without acknowledgement of other negative impacts or potential harms, including those on economy, employment, and mental health (New Zealand Government 2020). This impact was at times framed as ‘sacrifice’ (Malpass 2020; Mulgan 2020). Sacrifice is a flexible term of longstanding anthropological and sociological interest, applied here in relation to the cost (on freedoms of movement, socialisation, and economic productivity) borne by the majority, on behalf of a vulnerable minority. This is an example, as Roitman (2014) also discusses, of the way crisis frames can come with an imperative, engender sense of purpose. In this case the purpose depended on a newly (re)constituted category of ‘vulnerable people’ as a crucial part of the biopolitical assemblage of lockdown. The subject formation of many medical crowdfunders at this time (and their claims to care) depended on become one of ‘those’ people—but through publicly narrating themselves as such, rather than through government classification. These crowdfunding campaigns thus provide evidence of how the discourse of vulnerability worked, and was reworked, not only at the level of the populations, but also at the level of the self, as crowdfunders negotiated subjectivities defined by both the positive and negative potentialities of vulnerability.

Trundle et al. write that “illness and recovery involve multiple interconnected vulnerabilities at the somatic, social and political levels” (2018, p. 1). The written and visual storytelling practices through which crowdfunders wove the pandemic into their stories, often evoked all of these levels of vulnerability. They highlighted somatic vulnerability through vivid descriptions and visual images of pain and suffering, through discussing scarcity or lack of access to particular biotechnological objects (such as bone marrow or stem cells), and by referring to biological processes in relation to time (for example, cancerous cells spreading). They highlighted social vulnerability through detailing a lack of (or barriers to) social support, evoking affective dynamics of being “alone”, being scared, or missing loved ones, as well as mentioning fear around increased risk in public spaces, often due to lowered immune status (linking back to somatic vulnerability). They highlighted political vulnerability when they referred to border closures or restrictions around moving biological material internationally, as well as infrastructural changes or interruptions to healthcare delivery, since these were the result of political decisions.

The potent potential for crowdfunders using storytelling to publicly constitute themselves as vulnerable in COVID times, was to mobilise the already circulating discourses of self-sacrifice on behalf of vulnerable people and through this, to elicit responses of care—an example of “harnessing positive vulnerabilities in order to lessen the effects of damaging vulnerabilities” (Trundle et al. 2018, p. 2). There are similarities here with what others have written about the formation of health subjectivities in situations of crisis, including Petryna’s work on biological citizenship in the Ukraine after Chernobyl, where disabled identities became a survival strategy (2002, in Whyte 2009), and Trundle’s work on New Zealand nuclear test veterans, for whom vulnerability became “a positive means to reconceptualize the self, a strategy for gaining resources, a means to reconceptualize the body and its place in society, and a site for the exercise of contested power relationships” (Trundle et al. 2018, p. 13). For the crowdfunders I studied too, their vulnerability was not just ‘productive’ in that it *happened* to produce positive responses of care, but rather it was ‘strategic’—a position taken up deliberately as a strategy for securing care in a competitively marketized setting. It is also distinct in that it seeks to make claims for care and responsibility from the public, rather than the state, a point I come back to in the following section.

There was no single subject position among the crowdfunding recipients whose narratives I studied, and while their specific forms of vulnerability sometimes aligned with those in the category of ‘vulnerable people’ as defined by the government (as when they were immuno-compromised, or aged), at other times their vulnerability went beyond these, or represented different or competing vulnerabilities. Interestingly, it seemed many people were able to use this discourse of vulnerability as part of attempts to evoke care, even when the type of vulnerabilities they were experiencing were *not* those specifically flagged by the government, and in fact even when they were *made* vulnerable *by* the same government lockdown policies.

### Letting die: critique and resistance and care within systems

It is essential to consider the unspoken assumptions, the ambiguities, and the contradictions, underlying the lockdown as an exercise of biopower. New Zealand’s response to COVID-19 was heralded, internationally, as a success (Appleton, forthcoming). But while national pride swelled on official stages, a study of crowdfunding offers a more nuanced appreciation of the costs and difficulties of this achievement, and of the diverse experiences within it, particularly for those who were already precariously positioned—biologically, economically, politically—before the pandemic began. The ethics of care emphasises relationships of dependency (Baym 2015). This is arguably not only about dependency in interpersonal relationships, but also dependency in relationships between individuals and systems/structures. Indeed, practices of crowdfunding during COVID-19 seem to stand as accounts of these relationships just as much as interpersonal ones; accounts of the dependency of these precariously positioned individuals on healthcare systems that were suddenly restricted to them; and accounts of their increasing dependence on digital fundraising infrastructures and networked publics, as incomes dropped and in-person fundraising options dwindled. It is the disruption to, or breakdown of, these infrastructures that can render their political, ethical and relational features as more evident (Bowker et al. 2010), and thus more open to critique.

While particular vulnerable groups benefitted from the reduced spread of the virus that lockdown protocols supported, there *were* people whose health and wellbeing was adversely impacted by changes to healthcare delivery; people who bore a greater cost than most, and in fact were made vulnerable by the lockdown and its restrictions and interventions. Through the lens of biopower, the enforcement of the lockdown was both a refusal to ‘let die’, and an insistence to ‘make live’, for particular groups (Rabinow and Rose 2006). But other vulnerable groups experienced their right to life as threatened by the protocols that interrupted or delayed their treatments, diagnostic processes, or systems of social and economic support (Robinson and Wardell 2020). This is an example of competing vulnerabilities, and in fact structural violence, in that some people were actively prevented from meeting their health needs. Viewing these people’s (mediated, mediatised, and marketised) stories illustrates the “uneven effects of social conditions” (Whyte 2009, p. 6) on the formation of biopolitical and moral subjectivities.

In particular, it was difficult for the people *made* vulnerable (or more vulnerable) by lockdown, to navigate telling their stories in just the right way. The stories that included reference to the negative impacts of lockdown upon them can be read as expressing resistance in the way Rabinow and Rose discuss—where claiming a right to life, to one’s body, to health, and to the satisfaction of one’s needs, forms a sort of political struggle, and life becomes a “political object” (2006, p. 196). Indeed, in other settings, such as in the #Fight4OurHealth campaign in the USA (which addressed healthcare legislation and insurance precarity) the circulation of affecting illness narratives has been a key activist strategy (Merid 2019). But while sharing biographical narratives *can* be part of legitimate political action, empathy around others’ suffering does not always go hand in hand with structural critique, as Recuber’s (2015) study of “We are the 53%” campaign (which reacted against Occupy’s “We are the 99%” movement) showed. In fact, *none* of the stories I studied professed *overt* critique of lockdown protocols that had affected their access to healthcare. Rather they performed compliance to the affective regimes of New Zealand’s lockdown, checking in and rallying others to stick to lockdown protocol in a way that reads as very sincere—even while acknowledging themselves as suffering, disadvantaged, or vulnerable under these protocols.

The larger framing of ‘the times’ contributed to naturalising the specific political and moral characteristics of NZ’s COVID-19 response. The team of five million were asked to ‘unite again COVID’—a biological rather than political threat. In medical crowdfunding narratives, the impacts of lockdown protocols on patients’ healthcare experiences, were things that ‘happened’ as part of the crisis, rather than things that were ‘done to’, or choices that were made, masking the politico-economic characteristics of regulatory interventions. This was important because of the potential implications or backlash on those judged ‘noncompliant’ (Trnka 2020a). But also because, despite neoliberal rhetoric’s emphasis on individualism, societies continue to operate through a range of interpersonal, collective and state–citizen obligations, and the view of the state as “the final bastion of protection” may remain even when scepticism is present (Trnka and Trundle 2014; Trnka 2020a, p. 368). Thus, a part of the crowdfunders’ efforts to express a ‘correct’ subjectivity, involved discussing systemic and politico-economic contexts of their situation (to prove themselves blameless in their need) without threatening their position as a ‘good’ citizen, deserving of assistance, by critiquing those same systems.

Crowdfunding is a good example of how everyday practices can be part of enacting citizenship—a crucial dimension of social, political, and moral subjectivity, that involves both rights and duties —— as a mode of belonging. Neoliberal citizenship has its own characteristics, whereby the public is created as “co-responsible” for the public good (Muehlebach 2012, p. 8). This aligns with the logic of “networked responsibility” that underlies crowdfunding. In this study these crowdfunders active and public subjectification as good pandemic citizens worked to both support their claims of care from the ‘the crowd’, and to interpellate audiences into the same context-specific moral framework (of responsibility, kindness, sacrifice). They were thus active in reproducing the moral system while also remaking themselves strategically (and at an intimate level) within it.

## Conclusion

Even in states of emergency, measures to contain risk should not preclude public critique (Scarry 2010). We must stay wary of the “seduction to stop thinking”, especially in crises that are likely to be complex and evolving, rather than discretely solvable, and instead take up the responsibility of engaging in “how best to protect one another, both within and beyond national boundaries” while still fostering open-eyed and inclusive debate (Trnka, forthcoming, n.p.). Towards these goals, in this article I have examined the healthcare narratives of New Zealand medical crowdfunders, during and directly after the national lockdown. While acknowledging the way the campaigns narratives point to interrelated biological, social, and economic forms of precarity, the analysis has gone further, to raise questions about how citizens navigate uneven needs during uncertain times, through nuanced moral systems; how they are shaped by these contexts, but may also leverage them to meet their needs. As such my study affirms ‘the subject’ as

the site of experience, memory, storytelling and aesthetic judgement, an agent of knowing as much as of action; and the conflicted site for moral acts and gestures amid impossibly immoral societies and institutions.

(Biehl et al. 2007, p. 14).

Analysing medical crowdfunding narratives during COVID has functioned not only to bring a critical lens onto the varied and nuanced effects of the lockdown on New Zealand’s population, but also emphasises these people’s strategic self-positioning in relation to COVID-19 specific discourses around vulnerability, responsibility, and sacrifice, in attempts to secure care from the crowd. In this way, I have discussed how crowdfunding platforms, as one space for performing pandemic citizenship—one tool for the subjectification of recipients, their families, and their audiences—are generative spaces.

As lockdown intervened on ‘life itself’, crowdfunders narrated their health as entangled with wider systems while steering away from a critical position on these. They aligned themselves with affective regimes of kindness, solidarity and positivity in the ‘team of 5 million’. Through narrating both choice and constraint in a “scary world”, they took up positions as responsible, risk-sensitive citizens, while also strategically leveraging their vulnerability, against the category of ‘vulnerable people’ in efforts to secure care. As such, in answer to my research question, I have concluded that the subjectivities of medical crowdfunders in Aotearoa New Zealand *were* visibly shaped by the specific biopolitical regimes of New Zealand’s lockdown, as well as reflecting wider neoliberal moral economies that fed into and through pandemic discourses.

There are other angles into the experiences of medical crowdfunders, and other health subjects, during the pandemic and beyond, that access yet more intimate knowledge of these processes of subjectification. Interviews, longer-term case studies, and perhaps even autoethnographies could provide embodied insights. In addition, the timing of this study meant I also could not address the responses to, or outcomes of, the crowdfunding campaigns in this dataset, to know how successful the strategies described were. Future research may be able to more holistically situate ‘care’ and moral performance through examining the donor perspectives and practices as well.

Concluding her research on chemotherapy crowdfunding in the USA, just as I was beginning mine, Paust (2020, p. 100) writes that “ethnographic research at the intersection of health and economic precarity is needed now more than ever”—with the uneven effects of lockdown playing into existing social inequalities, and citizens negotiating with their own responses to the outworkings of biopower—including the possibilities and paradoxes of care between individuals, and between individuals and systems or infrastructures—under new biopolitical regimes. The crisis is ongoing, and as many countries continue to grapple with lockdowns and other forms of biopolitical intervention, as well as the devastating effects of the virus itself, social scientists will undoubtedly continue to play an important role in providing insight to the precarious world in which we must somehow live, as both products and agents of history (Biehl et al. 2007, p. 14).

## Footnotes

1 Defined as campaigns where the recipients were New Zealand citizens or residents, or where the creators where New Zealand citizens or residents and the recipient was overseas but New Zealand was explicitly mentioned in the campaign story.

2 Crowdfunding is an assemblage of people and technologies, with the majority of campaigns (Lukk et al. 2018; Wardell 2020) involving a family member or friend setting up the campaign on behalf of a recipient, who may or may not ‘ghost-write’ or contribute collaboratively to the page story and to subsequent campaign updates. In this article when I refer to ‘crowdfunders’ I include both the campaign creator/s and the campaign beneficiary as collaborators (see also: Neuwelt-Kearns 2020; Neuwelt-Kearns et al*.* forthcoming).

3 GoFundMe provided the figure of one third, in 2018, while Givealittle has suggested in private correspondence (Givealittle 2018, personal communication) that their health category comprised more than 43% of all donations—which may in reality be more, since many health-related campaigns are also found listed in other categories of the site (such as ‘Kiwi Kids’, ‘Challenges’ or ‘New Zealanders’).

4 Minimal campaign fundraising occurred for accidents, as the nation has a more comprehensive state insurance system for accident compensation and support during recovery. The campaigns that were related to accidents also less frequently requested funds for general living costs, than did those for illness or disability (Wardell 2020).

5 It is important to distinguish that a *biopolitical subjectivity* is not just a way of being in the world in relation to biology *and* politics, but rather it is one’s way of being in the world as constituted through or against strategic, bureaucratic interventions on life and health.
